# Middle Turbinectomy in Endoscopic Endonasal Skull Base Surgery: How Significant Is Its Impact on Quality of Life?

**DOI:** 10.3390/curroncol33070423

**Published:** 2026-07-15

**Authors:** Narin Nard Carmel Neiderman, Orr Raved, Harel Sofer, Idan Peled, Idan Ben Nachum, Ran Bilaus, Tomer Ziv Baran, Omer J. Ungar, Lior Gonen, Avraham Abergel

**Affiliations:** 1 Department of Otolaryngology, Head and Neck Surgery and Maxillofacial Surgery, Tel Aviv Sourasky Medical Center, Affiliated to Tel Aviv University, Tel Aviv 6423906, Israel; orr.raved@gmail.com (O.R.); harel.sofer@mail.huji.ac.il (H.S.); idanpel@clalit.org.il (I.P.); idanben@tlvmc.gov.il (I.B.N.);; 2 Department of Epidemiology, Grey Faculty of Medicine, Tel Aviv University, Tel Aviv 6997801, Israel; 3 Department of Neurosurgery, Tel Aviv Sourasky Medical Center, Tel Aviv 6423906, Israel

**Keywords:** quality of life, endoscopic endonasal sinus surgery, pituitary adenoma, skull base, middle turbinate resection, ASBS-Q, SNOT-22

## Abstract

Background: Endoscopic endonasal approaches for pituitary adenomas are associated with improved clinical and quality of life (QOL) outcomes. However, the necessity of middle turbinate resection to optimize surgical exposure remains controversial, particularly regarding its potential impact on postoperative nasal and tumor-related QOL. Methods: This prospective cohort study included adult patients undergoing endoscopic endonasal transsphenoidal resection of pituitary adenomas between 2014 and 2021 at a tertiary center. Patients were divided into those undergoing bilateral middle turbinectomy and those with turbinate preservation. Tumor-related QOL was assessed using the Anterior Skull Base Disease-Specific Questionnaire (ASBS-Q), and nasal QOL using the Sinonasal Outcome Test-22 (SNOT-22), at baseline and multiple postoperative time points up to >6 months. Clinical and surgical variables were collected and compared between groups. Results: Our study included 73 patients, 51 (69.9.%) of whom underwent middle turbinate resection and 22 (30.1%) who did not. The difference in overall ASBS-Q scores did not alter significantly between both groups during the long-term postoperative course (>6 months). SNOT-22 score differences also did not alter significantly throughout the entire postoperative course. Conclusion: Middle turbinectomy during endoscopic endonasal resection of pituitary adenomas was not associated in our cohort to adversely affect long-term nasal or tumor-related QOL.

## Simple Summary

Endoscopic surgery through the nose is commonly used to remove pituitary tumors, but surgeons often debate whether removing a normal nasal structure called the middle turbinate is necessary or may harm patients. In this study, we evaluated 73 patients who underwent this surgery, comparing those who had the turbinate removed with those in whom it was preserved. We assessed both nasal symptoms and overall well-being using patient-reported questionnaires over time. Our results showed that removing the turbinate did not worsen long-term quality of life related to either nasal function or the tumor. Short-term changes were temporary and resolved over time.

## 1. Introduction

The endoscopic approach to the anterior skull base has revolutionized pituitary gland surgery [1]. This technique offers several advantages, including shorter hospitalization, reduced morbidity and discomfort, lower rates of postoperative complications, and improved overall survival [2,3]. However, utilizing a healthy nasal corridor for surgical access may result in unintended postoperative sequelae. Iatrogenic sinonasal complications, such as crusting, rhinosinusitis, epistaxis, and hyposmia, are not uncommon and can contribute to a considerable postoperative disease burden [1,4,5,6,7,8,9,10].

Many surgeons routinely resect one or both middle turbinates in order to enhance exposure and facilitate surgical access during endoscopic skull base surgery [11]. Middle turbinectomy is also commonly performed during functional endoscopic sinus surgery (FESS), particularly in cases of chronic rhinosinusitis with nasal polyposis [12], with some studies reporting beneficial effects, such as improved olfactory function and reduced perioperative stress response [13]. However, unlike FESS, surgery for sellar lesions typically involves traversing a healthy nasal cavity. In this context, resection of the middle turbinate—although intended to improve access—may paradoxically impair nasal function. Reported consequences include altered airflow and drainage patterns, olfactory dysfunction, and delayed postoperative recovery [14,15].

In 2020, Wong et al. showed that middle turbinate resection did not correlate with poorer postoperative nasal obstruction, sinonasal function, or sleep quality among 294 patients operated for non-sinonasal pathologies [1]. These results were further strengthened in 2021 by Tao et al. [2], who showed that the Sinonasal Outcome Test (SNOT-22) and visual analog scale (VAS) for olfactory findings did not deteriorate among 75 patients operated on for pituitary adenomas [2]. Tumor-specific-related QOL, however, has seldom been compared between patients with and without middle turbinate resection [3].

In this study, we aimed to strike a balance between achieving effective tumor resection and preserving nasal function. Specifically, we sought to compare both tumor-related and nose-related quality of life (QOL) in patients undergoing endoscopic endonasal resection of sellar tumors with or without preservation of the middle turbinate. By evaluating whether middle turbinate preservation influences postoperative QOL outcomes, we reasoned that our findings may add to the growing evidence base regarding the role of middle turbinectomy in skull base surgery.

## 2. Materials and Methods

### 2.1. Patient Population

All adult patients who underwent endoscopic endonasal resection of the anterior and middle skull base due to pituitary adenomas at our institution between 2014 and 2021 were enrolled in the study. They were asked to complete QOL questionnaires as part of their pre- and postoperative evaluations. The preoperative workup included a comprehensive ophthalmologic evaluation, an endocrine laboratory assessment, and imaging studies with both computed tomography (CT) and magnetic resonance imaging (MRI).

Prior to surgical intervention, all patients were evaluated by a multidisciplinary team consisting of neurosurgeons, endocrinologists, and otolaryngologists specializing in rhinology. The same surgical team also performed all surgical procedures. In accordance with our institutional policy, radiation therapy was offered as an alternative treatment for patients deemed unsuitable candidates for endoscopic surgery and for those in whom previous endoscopic surgery had failed. This prospective study was approved by the Institutional Ethics Committee (TLV-0761–19).

The study population was stratified into subgroups based upon tumor characteristics, tumor size, presence of intraoperative cerebrospinal fluid (CSF) leak, and the reconstruction techniques employed in order to identify factors associated with poor postoperative QOL. Tumor size was determined by preoperative MRI and classified as microadenoma (<1 cm), macroadenoma (>1 cm), or giant adenoma (>2.5 cm). Histopathological confirmation was obtained for all lesions, and patients with non-pituitary adenomas were excluded from the study. Tumors were further categorized as either secreting or non-secreting. Surgical success was defined according to tumor type: specifically, the achievement of gross total resection (GTR) for non-secreting tumors or those exerting a mass effect and as the achievement of endocrine remission for secreting tumors.

Inclusion criteria for the study were age >18 years, the ability to provide informed consent, and the ability to complete the study questionnaires. Exclusion criteria were a medical history of significant nasal pathology (e.g., nasal cavity tumors), non-sellar lesions, posterior or lateral skull base pathology, pregnancy, age <18 years, inability to provide informed consent, inability to complete the questionnaires, or insufficient questionnaire responses (defined as completion of less than 75% of items), as well as refusal to participate. Follow-up was conducted in the outpatient clinic at 3 postoperative intervals: 2 months after surgery, and between 2 and 6 months postoperatively. Patients who did not complete at least the 2-month follow-up were excluded from the study.

### 2.2. QOL Assessment and Questionnaire

The psychological, social, and physical well-being of the patients were assessed via the Anterior Skull Base Disease-Specific QOL Questionnaire (ASBS-Q), a disease-specific multidimensional questionnaire designed for patients undergoing surgery due to tumors involving the anterior skull base [4] All patients were required to complete the ASBS-Q at the above-mentioned postoperative intervals. The questionnaire is a patient-based measurement designed for self-administration. It consists of 6 domains, the role of performance (8 items), physical function (7 items), vitality (6 items), pain (3 items), specific symptoms (7 items), and emotional state (5 items), all of which yield a total of 36 questions with a nominal scale of 5 steps for each question. A higher score reflects a higher satisfaction regarding tumor-related QOL. All questions have an identical level of importance. The domain of specific symptoms includes 7 questions on several aspects that are most relevant to this patient population: altered taste, smell, and appearance, as well as epiphora, nasal secretions, and visual disturbances [5].

Patients also completed the Hebrew-validated version of the Sinonasal Outcome Test-22 (He-SNOT-22) questionnaire [16]. This self-administered, patient-reported instrument was used to assess nose-related QOL at the specified postoperative intervals. The total score ranges from 0 to 110, with higher scores indicating greater impairment in nasal function and QOL. The questionnaire was administered once preoperatively and again between 2 and 6 months postoperatively.

### 2.3. Surgical Technique

Tumor extirpation was performed by means of the expanded endoscopic endonasal approach in all patients. The cohort was divided into 2 groups, one comprising those who underwent bilateral middle turbinectomy and the other comprising patients in whom the middle turbinates were preserved. Both turbinates were removed to facilitate a bi-manual approach in cases where a middle turbinectomy was performed. The middle turbinate was fully resected in majority of cases, using a combination of endoscopic scissors, through-cutting forceps, and microdebriders; emostasis and treatment of residual mucosal attachments were completed using suction Bovie and bipolar cautery. Until 2018, bilateral middle turbinectomy was routinely performed as part of the surgical approach. From 2018 onward, middle turbinectomy was performed selectively following intraoperative discussion with the neurosurgical team, when a wider surgical corridor was deemed necessary. Indications included a narrow nasal cavity with small ethmoid sinuses, a prominent or bullous middle turbinate, or the presence of a firm, non-suctionable macroadenoma.

In order to create a wide surgical corridor, a partial anterior and posterior ethmoidectomy was performed on the right nasal cavity, allowing space for both the endoscope and suction instruments. Resection of the inferior posterior third of the superior turbinate was also performed when a supra-sellar approach was required.

A nasoseptal flap (NSF) was harvested at the beginning of the procedure in cases where a high-flow cerebrospinal fluid (CSF) leak was anticipated such as in cases involving a supra-sellar extension. The surgical corridor was then completed with a wide sphenoidotomy following partial posterior septectomy and vomer resection, which lowered the anterior wall of the sphenoid sinus. During dissection of the posterior septal mucosa from the vomer, efforts were made to preserve the posterior septal artery by reflecting the mucosa of the posterior septum and choana downward, thereby creating a small “rescue flap.” This technique enabled flap elevation later in the surgery if an unanticipated high-flow CSF leak had occurred.

Reconstruction was customized based upon the size of the defect and the CSF leak status. An NSF that had been prepared in advance was used in conjunction with autologous fat when a high-flow CSF leak was anticipated, in the presence of very large supra-sellar tumors, or proximity of the tumor to the third ventricle. Autologous fat alone was used for reconstruction in cases with low-flow CSF leaks [15,17].

### 2.4. Statistical Analysis

Categorical variables were described as frequency and percentage. Continuous variables were reported as mean and standard deviation. The overall preoperative and postoperative score as well as scores of selected questions were compared by the Wilcoxon signed-rank test. Comparisons between patients with secreting and non-secreting tumors, between patients with and without endocrine remission, and between patients with and without GTR were performed with the Mann–Whitney test for the continuous variables and the chi-square test or Fisher’s exact test for the categorical variables. All statistical tests were 2-sided and a *p*-value < 0.05 was accepted as statistically significant. SPSS software (IBM SPSS Statistics for Windows, version 25, IBM corp., Armonk, NY, USA, 2017) was employed for all statistical analyses. Linear mixed models were applied for repeated measurements analysis in order to compare the change in SNOT22 and ASBQ between the two study groups while controlling for potential confounders. A first-order auto regression was used for repeating measurements and interaction between study group and time points were used to study the difference in each time point between the two groups.

## 3. Results

A total of 73 consenting patients met the inclusion criteria and participated in the study. The mean age of the cohort was 52.92 ± 16.86 years, and 42 (57.5%) were males (Table 1). The major comorbidities consisted of hyperlipidemia in 30 patients (41.1%) followed by essential hypertension in 29 (39.7%) patients. The patients’ clinical data are shown in Table 1. The most common initial presentation was endocrine impairment, which was found in 49 patients (67.1%). It was followed by preoperative headaches in 36 patients (50%). Visual impairments associated with skull base pathology were diagnosed in 30 patients (41.1%) The average size of the largest tumors depicted in preoperative MRI studies was 21.27 ± 14.24 mm (Table 1).

The majority of patients in our cohort underwent middle turbinectomy (n = 51, 69.9%). Seventeen procedures (23.3%) were revision surgeries, reflecting our institution’s role as a tertiary referral center. Intraoperative CSF leaks occurred in 25 cases (34.2%), of which nine (36%) were classified as high-flow leaks. An NSF was utilized for reconstruction in 25 patients (34.7%), accounting for 45.5% of all cases in which reconstruction was performed (Table 1).

**Figure 1:**
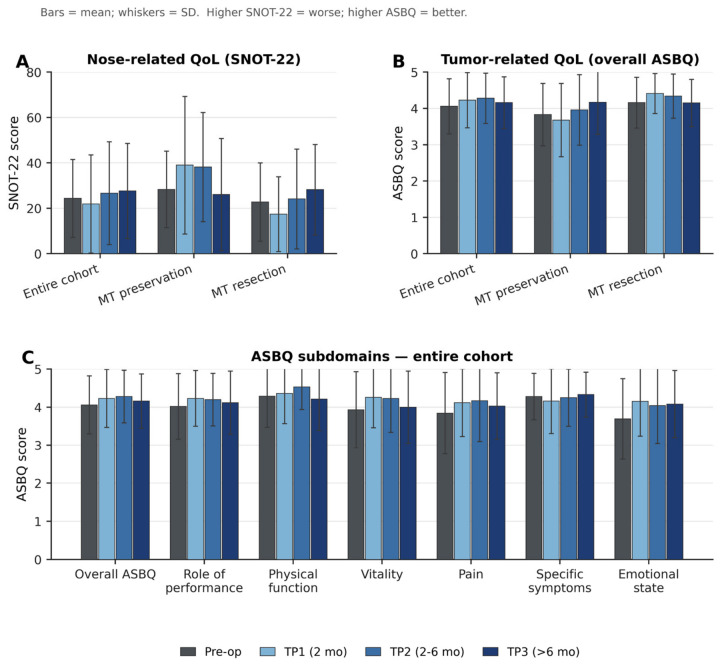
Nasal and tumor-related QOL among patients with and without middle turbinectomy (n = 73). (**A**) SNOT22 scores among entire cohort and patients with and without middle turbinectomy; (**B**) ASCQ total score among entire cohort and patients with and without middle turbinectomy; (**C**) ASCQ subdomains’ score among entire cohort. M = months; Op = operation.

The response rate for the questionnaires at the first postoperative follow-up (2 months after surgery) was 82.1% (n = 60), and this rate remained consistent at the subsequent follow-up intervals (2–6 months and beyond 6 months postoperatively). Notably, both nasal and tumor-related QOL scores remained relatively high throughout the follow-up period (Table 2, Figure 1).

Comparisons of the baseline characteristics between patients who underwent middle turbinectomy and those who did not revealed no significant differences in medical comorbidities or initial clinical presentation, except for the presence of obstructive sleep apnea (OSA) and tumor size on imaging studies (Table 3). Patients in the turbinectomy group had significantly larger tumors (23.78 ± 15.64 mm vs. 15.56 ± 8.09 mm; *p* = 0.039). Additionally, the use of a nasoseptal flap for reconstruction was significantly more common among patients who underwent middle turbinectomy (n = 25, 50.0%) compared to those with preserved turbinates (n = 0, 0%; *p* = 0.001). While overall surgical success rates did not differ significantly between groups, a higher proportion of patients in the turbinectomy group achieved endocrine remission (see definition in materials and methods), (n = 26, 63.4%) compared to those without turbinectomy (n = 5, 33.3%; *p* = 0.045). Furthermore, pneumocephalus occurred significantly less frequently in the turbinectomy group (0% vs. 20%; *p* = 0.031). No significant differences were found in the rates of other major complications between the 2 groups (Table 3).

**Figure 2:**
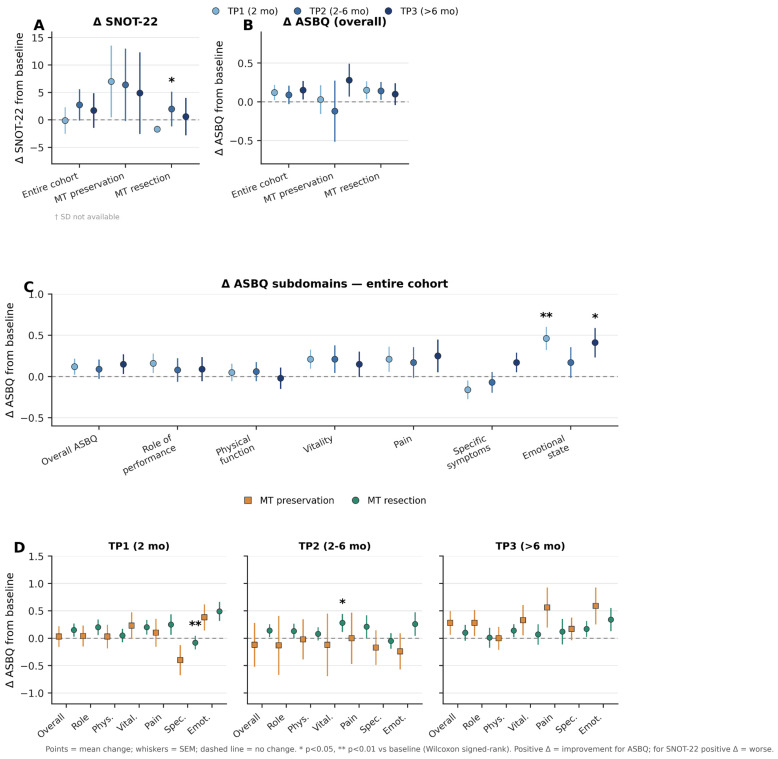
QOL score difference between preop and three different time points (n = 73). (**A**) SNOT22 scores difference from baseline among entire cohort and patients with and without middle turbinectomy. (**B**) ASCQ score difference among entire cohort and patients with and without middle turbinectomy. (**C**) ASBQ subdomain score difference among entire cohort. (**D**) ASBQ subdomains among patients with and withour middle turbinectomy. MTR = middle turbinate resection; MTP—middle turbinate preservation; M = months; Op = operation.

Comparisons between the baseline (preoperative) QOL scores between patients with and without middle turbinate resection revealed no significant differences in the overall ASBS-Q or SNOT-22 scores (Figure 1). Interestingly, however, the vitality subdomain of the ASBS-Q was significantly higher in the middle turbinectomy group (4.07 ± 0.94) compared to the turbinate preservation group (3.61 ± 1.08; *p* = 0.049) (Table 4, Figure 2).

Also interesting is that patients who underwent middle turbinate preservation reported decreased QOL across all tumor-related domains. As shown in Table 4, they had significantly lower overall ASBS-Q and subdomain scores compared to those who underwent turbinectomy (Table 4, Figure 1). The SNOT-22 scores were borderline significantly lower in the turbinate preservation group, suggesting slightly better short-term nose-related QOL However, at the subsequent follow-up intervals (2 to 6 months and beyond 6 months), there were no significant group differences in either tumor-related or nose-related QOL, indicating that the initial decrease was a transient effect (Figure 2).

To further investigate the observed trends, we conducted a score difference analysis by comparing each patient’s preoperative QOL scores to those at the three postoperative time points in both groups (Table 5). This analysis revealed that there were no significant differences across the entire follow-up period relative to the baseline scores (Table 5 and Table 6). Furthermore, when comparing QOL scores to the preoperative baseline across all three postoperative time points between patients who underwent turbinectomy and those who did not, the only significant improvement was observed in the emotional subdomain of the ASBS-Q at the first follow-up (Table 5, Figure 2).

To control for possible confounders, while investigating the change in SNOT and ABSQ between middle turbinate resection and preservation in each time point, linear mixed models were applied to adjust for age, gender, CSF leak intraoperatively, secreting or non-secreting adenoma and tumor size. In all comparisons, there was no statistically significant difference (Table 7).

## 4. Discussion

We compared tumor- and nose-related QOL of 73 patients who underwent endoscopic endonasal resection of pituitary adenomas via the extended endoscopic endonasal approach with or without middle turbinate preservation. In our prospective non-interventional cohort, the addition of a middle turbinectomy was not associated with any deterioration of QOL. While there was a slight decrease in crude tumor-related QOL at the 2-month postoperative follow-up among the patients who underwent middle turbinate preservation, the difference was not significant when score differences between groups were evaluated, nor when the patients’ preoperative baseline scores were compared (Table 5 and Table 6). Furthermore, tumor-related QOL in our cohort normalized in both the intermediate (2–6 months) and long-term (>6 months) follow-up periods, indicating that we did not find a significant deterioration in QOL was observed over time.

Endoscopic skull base surgery changes nasal anatomy to the extent that nasal airflow is significantly altered postoperatively [6]. Middle turbinate resection increases airflow in the middle meatus and reduces airflow in the superior and inferior meatus [6]. Middle turbinate resection was found to significantly influence airflow distribution between the bilateral nasal cavities and the different parts of the nasal cavity in computational fluid dynamics. Moreover, changes in temperature and humidity were highly affected by middle turbinate resection [7].

Given our above findings, an increase in SNOT-22 scores following middle turbinectomy might have been anticipated. However, they align with those of Wang and Tae [2,8], reinforcing the conclusion that although a middle turbinectomy may alter nasal airflow in a healthy nose, it does not result in deterioration of patient-reported nasal QOL. Similarly, studies by Conrad et al. [9] and Baban et al. [10] demonstrated that middle turbinate preservation during resection of benign neoplasms did not significantly impact nose-related QOL at 6 months postoperatively. However, those studies did not evaluate the potential benefits of middle turbinectomy in terms of surgical complications, particularly in patients with adenoma.

The higher endocrine remission rates observed in the turbinectomy cohort should be interpreted with caution, as this finding may reflect differences in tumor characteristics, surgical exposure, and operative strategy rather than a direct effect of the improved exposure attributed to middle turbinectomy itself. Given the non-randomized nature of the study and the selective use of turbinectomy in more complex cases requiring a wider surgical corridor, potential confounding factors may have influenced endocrine outcomes independently of turbinectomy.

Although complications were not the primary focus of our study, we observed no significant difference in the rate of major postoperative complications between the turbinectomy and the turbinate preservation groups. This supports the notion that the performance of a middle turbinectomy does not compromise patient safety. Moreover, this finding is consistent with the work of Nyquist et al. [11], who demonstrated that middle turbinate preservation is feasible in most endoscopic endonasal transsphenoidal skull base surgeries while still allowing for adequate exposure and successful tumor resection and reconstruction.

The olfactory epithelium is known to reside not only within the superior nasal cavity but also along the medial surface of the middle turbinate [12]. As such, endoscopic biopsies of the olfactory mucosa—including those from the middle turbinate—have been recognized as a viable extracranial source of neural progenitor cells. Based upon these anatomical and functional considerations, we had initially hypothesized that a middle turbinectomy might negatively affect tumor-related QOL, particularly in domains related to special sensory functions such as olfaction. However, our current findings did not support this hypothesis. Mariano et al. investigated the impact of partial middle turbinectomy in patients treated for nasal obstruction and found no clinically significant effect on olfactory function [13]. Similarly, Ting et al. demonstrated that partial middle turbinate resection during endoscopic endonasal approaches did not impair olfaction [14]. While those studies support the functional safety of partial turbinectomy, our literature review did not reveal any comprehensive analysis specifically addressing the consequences of complete middle turbinectomy on olfactory outcomes.

We observed a slight deterioration in tumor-related QOL scores, as measured by the ASBS-Q, during the early postoperative period. However, this decline was transient and did not reach a level of significance. In addition to the nasal QOL, our study assessed skull base tumor-specific QOL outcomes using the ASBS-Q. Notably, the short-term decline in QOL appeared to be most pronounced within the emotional burden subdomain, reflecting the psychological burden experienced by patients in the immediate postoperative phase. Similar findings were reported in previous studies conducted by our group [15,18]. These emotional and overall QOL scores improved and returned to baseline levels over the course of long-term follow-up in the majority of patients.

This study has several limitations. It was a single-center prospective observational cohort with a limited and unequally distributed sample, without randomized allocation. The study was not designed or powered as an equivalence or non-inferiority analysis; therefore, the absence of statistically significant differences should not be interpreted as proof of equivalence or exclusion of clinically meaningful differences. Patients undergoing middle turbinectomy had larger tumors and more frequently required nasoseptal flap reconstruction, reflecting greater surgical complexity and introducing important selection bias. Although adjusted linear mixed-model analyses accounted for age, sex, tumor size, adenoma secretory status, and intraoperative CSF leak, residual confounding remains likely. In particular, nasoseptal flap reconstruction could not be meaningfully adjusted for because it occurred exclusively in the turbinectomy group. Although we anticipated that more complex patients will suffer from decreased QOL, it did not reflect QOL outcomes in our cohort. Moreover, preservation of the middle turbinate may extend surgery length, and that parameter was not included as an outcome in our analysis.

Other potentially relevant factors, including endocrine remission, postoperative pituitary dysfunction, revision surgery, baseline sinonasal disease, and temporal changes in surgical practice, may also have influenced patient-reported outcomes. Attrition at later follow-up may have introduced additional response bias.

Our study is also limited with a selection and fatigue bias and a significant dropout rate at the late follow-up point. We also included both secreting and non-secreting tumors. Another limitation is that adhesions were not scored postoperatively but rather assessed solely in the physical examination. While similar trends were observed in broader cohorts from our institution that included additional benign anterior skull base tumors, the relatively small number of non-adenomatous lesions limited robust subgroup analysis. Therefore, the present study was limited to pituitary adenomas to maintain cohort homogeneity and improve interpretability of the findings. As a result, the generalizability of these results to other benign anterior skull base pathologies may be limited. Finally, we did not routinely perform objective assessments of either nasal airflow or olfaction objective assessment. We used validated questionnaires that may not be sufficiently focused on specific domains, such as olfaction, to detect more subtle impairments in quality of life.

## 5. Conclusions

In this prospective real-world cohort, middle turbinectomy during endoscopic resection of pituitary lesions was not associated with statistically significant worsening of sinonasal quality of life or long-term tumor-related quality of life. These findings should be interpreted cautiously in light of the non-randomized group allocation, baseline differences in surgical complexity, and limited sample size, which preclude definitive conclusions regarding equivalence between the two strategies.

## Figures and Tables

**Table 1:** Cohort Characteristics of Patients operated in an extended endonasal approach for Tumor extirpation from anterior and middle skull base (n = 73).

Parameter (n = 73)	N (%)
Sex (male)	42 (57.5%)
Age (years)	52.92 ± 16.86 56 [37.5–66]
Comorbidities and medical history	
Diabetes mellitus	14 (19.2%)
Essential hypertension	29 (39.7%)
Dyslipidemia	30 (41.1%)
Other malignancy	5 (6.8%)
Cerebrovascular accident	3 (4.1%)
Ischemic heart disease	9 (12.3%)
Cardiac arrythmia	4 (5.5%)
Obstructive sleep apnea	5 (6.8%)
ASA score (>3) (n = 65)	22 (33.8%)
Initial presentation and imaging characteristics	
Microadenoma (<1 cm)	17 (23.3%)
Macroadenoma (>1 cm)	56 (76.7%)
Tumor largest diameter (mm) (n = 72)	21.27 ± 14.24 20 [9–29.5]
Preoperative visual impairment	30 (41.1%)
Preoperative headache (n = 72)	36 (50%)
Preoperative endocrine disorder	49 (67.1%)
Pan hypopituitarism	8 (11%)
Cushing syndrome	17 (23.3%)
Acromegalia	11 (15.1%)
Hyperprolactinemia	7 (9.6%)
Hypogonadism	7 (9.6%)
Hypothyroidism	8 (11%)
Hypocortisolism	2 (2.7%)
Surgery characteristics	
Revision surgery	17 (23.3%)
Middle turbinectomy	51 (69.9%)
Intraoperative CSF leak	25 (34.2%)
Low-flow CSF leak	16 (64%)
High-flow CSF leak	9 (36%)
NSF reconstruction (n = 72)	25 (34.7%)
Histopathological diagnosis	
Overall secreting tumor rate	29 (39.7%)
Corticotroph adenoma	19 (26%)
Null cell adenoma	26 (35.6%)
Somatotroph adenoma	12 (16.4%)
Lactotroph adenoma	4 (5.5%)
Gonadotroph adenoma	12 (16.4%)
Postoperative course	
Major complications	5 (6.8%)
Meningitis	1 (1.4%)
Postoperative CSF leak	2 (2.7%)
Postoperative CSF leak requiring surgical repair	2 (100%)
Intracranial hemorrhage	1 (1.4%)
Postoperative permanent DI (n = 72)	2 (2.8%)
Steroid-dependent (>6 months) (n = 69)	8 (11.6%)
Postoperative transient DI (n = 72)	7 (9.7%)
Major eye impairment (n = 69)	0 (0%)
Pulmonary emboli (n = 69)	0 (0%)
Cranial nerve impairment (n = 70)	1 (1.4%)
Pneumocephalus (n = 70)	3 (4.3%)
Minor complications	
Sinusitis (n = 67)	6 (9%)
Epiphora (n = 69)	0 (0%)
Iatrogenic septal perforation (n = 69)	3 (4.3%)
Deep vein thrombosis (n = 69)	0 (0%)
Adhesions required under local adhesiolysis (n = 66)	43 (65.2%)
Adhesions surgically treated (n = 66)	0 (0%)
Surgical outcomes	
Postoperative endocrine status unchanged (n = 72)	47 (65.3%)
Postoperative endocrine status improvement (n = 72)	22 (30.6%)
Postoperative endocrine status worsening (n = 72)	3 (4.2%)
Postoperative visual status unchanged	47 (64.4%)
Postoperative visual status improvement	19 (26%)
Postoperative visual status worsening	1 (1.4%)
No smell impairment (>6 months)(n = 69)	61 (88.4%)
Hyposmia (>6 months) (n = 69)	7 (10.1%)
Anosmia (>6 months) (n = 69)	1 (1.4%)
Surgical outcomes–technical	
Surgical success (n = 71)	42 (65.6%)
Achieved gross tumor resection (n = 64)	31 (55.4%)
Achieved endocrine remission (n = 56)	42 (59.2%)
Adjuvant treatment	
Irradiation treatment (n = 71)	6 (8.5%)
Overall Gy dosage (n = 5)	27.2 ± 5.4 26 [23–32]

ASA = American Society of Anesthesiology; mm = millimeter; NSF = nasoseptal flap; CSF = cerebrospinal fluid; cm = centimeter; CSF = cerebrospinal fluid; DI = diabetes insipidus. Categorical parameters are presented as N(%); continuous parameters are presented as mean ± standard deviation and median (50%) [interquartile range, IQR [25–75%]].

**Table 2:** Nose- and tumor-related QOL among pituitary adenoma patients over time (n = 73).

Parameter (n = 73)	Questionnaire Score (mean ± STD; Med [IQR])
Preoperative questionnaire scores	
Nose-related QOL: SNOT-22	24.34 ± 17.18 19.00 [10.00–38.00]
Tumor-related QOL: Overall ASBQ	4.06 ± 0.76 4.17 [3.66–4.68]
ASBQ subdomains	
Role of performance	4.02 ± 0.86 4.17 [3.57–4.71]
Physical function	4.29 ± 0.82 4.50 [3.86–5.00]
Vitality	3.93 ± 1.00 4.17 [3.50–4.67]
Pain	3.84 ± 1.07 4.00 [3.00–4.67]
Specific symptoms	4.28 ± 0.61 4.43 [4.00–4.71]
Emotional state	3.69 ± 1.06 3.80 [2.70–4.60]
2-month postoperative (TP1) questionnaire scores	
Nose-related QOL: SNOT-22 (n = 55)	21.92 ± 21.60 12.50 [6.00–33.50]
Tumor-related QOL: Overall ASBQ (n = 60)	4.23 ± 0.76 4.51 [3.65–4.82]
ASBQ subdomains	
Role of performance (n = 60)	4.23 ± 0.73 4.43 [3.67–4.86]
Physical function (n = 60)	4.36 ± 0.79 4.75 [3.75–5.00]
Vitality (n = 60)	4.26 ± 0.80 4.67 [3.67–4.83]
Pain (n = 59)	4.12 ± 0.89 4.33 [3.67–5.00]
Specific symptoms (n = 59)	4.16 ± 0.85 4.43 [3.71–4.86]
Emotional state (n = 59)	4.15 ± 0.91 4.40 [3.60–4.80]
2-to-6-month postoperative (TP2) questionnaire scores	
Nose-related QOL: SNOT-22 (n = 39)	26.68 ± 22.66 21.00 [8.00–47.50]
Tumor-related QOL: Overall ASBQ (n = 40)	4.28 ± 0.69 4.43 [3.91–4.84]
ASBQ subdomains	
Role of performance	4.20 ± 0.69 4.24 [3.85–4.86]
Physical function	4.53 ± 0.59 4.69 [4.34–5.00]
Vitality	4.23 ± 0.89 4.50 [3.92–5.00]
Pain	4.17 ± 1.07 4.67 [3.67–5.00]
Specific symptoms	4.25 ± 0.75 4.57 [3.86–4.79]
Emotional state	4.04 ± 0.99 4.40 [3.30–4.80]
6-month-and-onward (TP3) postoperative questionnaire scores	
Nose-related QOL: SNOT-22 (n = 43)	27.65 ± 20.98 25.00 [9.50–44.50]
Tumor-related QOL: Overall ASBQ (n = 40)	4.16 ± 0.71 4.33 [3.60–4.83]
ASBQ subdomains	
Role of performance	4.12 ± 0.83 4.43 [3.36–4.85]
Physical function (n = 60)	4.21 ± 0.82 4.50 [3.63–5.00]
Vitality (n = 60)	4.00 ± 0.95 4.50 [3.42–4.75]
Pain (n = 59)	4.03 ± 0.87 4.33 [3.42–4.67]
Specific symptoms (n = 59)	4.33 ± 0.59 4.57 [3.86–4.86]
Emotional state (n = 59)	4.08 ± 0.88 4.20 [3.60–4.90]

Categorical parameters are presented as N(%); continuous parameters are presented as mean ± standard deviation; median (50%) [interquartile range, IQR [25–75%]]. SNOT-22: sinonasal quality of life tool; ASBQ: anterior skull base questionnaire.

**Table 3:** Comparison of patient characteristics and outcomes between those who underwent middle turbinate resection vs. middle turbinate preservation (n = 73).

Parameter (n = 73)	Middle Turbinate Preservation (n = 22)	Middle Turbinectomy (n = 51)	*p*-Value
Sex (male)	13 (59.1%)	29 (56.9%)	0.860
Age (years)	58.18 ± 17.68 61.50 [48.75–70.25]	50.65 ± 16.14 47 [37–64]	0.081
Comorbidities and medical history			
Diabetes mellitus	4 (18.2%)	10 (19.6%)	>0.999
Essential hypertension	10 (45.5%)	19 (37.3%)	0.511
Dyslipidemia	12 (54.55)	18 (35.3%)	0.125
Other malignancy	1 (4.5%)	4 (7.8%)	>0.999
Cerebrovascular accident	0 (0%)	3 (5.9%)	0.549
Ischemic heart disease	4 (18.2%)	5 (9.8%)	0.439
Cardiac arrythmia	2 (9.1%)	2 (3.9%)	0.579
Obstructive sleep apnea	4 (18.2%)	1 (2%)	**0.027**
ASA score (>3) (n = 65)	10 (45.5%)	12 (27.9%)	0.313
Initial presentation and imaging characteristics			
Microadenoma (<1 cm)	5 (22.7%)	12 (23.5%)	0.941
Macroadenoma (>1 cm)	17 (77.3%)	39 (76.5%)	0.941
Tumor largest diameter (n = 72) in cm	15.56 ± 8.09 15.50 [7.75–22.25]	23.78 ± 15.64 22 [12–33.5]	**0.039**
Visual impairment	7 (31.8%)	23 (45.1%)	0.290
Headache (n = 72)	12 (57.1%)	24 (47.1%)	0.437
Endocrine status	14 (63.6%)	35 (68.6%)	0.677
Pan hypopituitarism	3 (13.6%)	5 (9.8%)	0.691
Cushing syndrome	4 (18.2%)	13 (25.5%)	0.498
Acromegalia	3 (13.6%)	8 (15.7%)	>0.999
Hyperprolactinemia	2 (9.1%)	5 (9.8%)	>0.999
Hypogonadism	2 (9.1%)	5 (9.8%)	>0.999
Hypothyroidism	3 (13.6%)	5 (9.8%)	0.691
Hypocortisolism	1 (4.5%)	1 (2%)	0.515
Surgery characteristics			
Revision surgery	5 (22.7%)	12 (23.5%)	0.941
Intraoperative CSF leak (n = 26)	4 (18.2%)	21 (41.2%)	0.057
Low-flow CSF leak	4 (100%)	12 (54.5%)	0.374
High-flow CSF leak	0 (0%)	9 (40.9%)	0.374
NSF reconstruction (n = 72)	0 (0%)	25 (50%)	**<0.001**
Histopathological diagnosis			
Overall secreting tumor	8 (36.4%)	21 (41.2%)	0.700
Postoperative course			
Major complications	1 (4.5%)	4 (7.8%)	>0.999
Meningitis	0 (0%)	1 (2%)	>0.999
CSF leak	1 (4.5%)	1 (2%)	0.515
CSF leak requiring surgical repair (n = 4)	1 (100%)	1 (33.3%)	>0.999
Intracranial hemorrhage	0 (0%)	1 (2%)	>0.999
Permanent DI (n = 72)	0 (0%)	2 (4%)	>0.999
Steroid-dependent (>6 months) (n = 69)	3 (13.6%)	5 (10.6%)	>0.999
Transient DI (n = 72)	1 (4.5%)	6 (12%)	0.427
Major eye impairment (n = 69)	0 (0%)	0 (0%)	
Pulmonary emboli (n = 69)	0 (0%)	0 (0%)	
Cranial nerve impairment (n = 70)	0 (0%)	1 (2.1%)	>0.999
Major pneumocephalus (n = 70)	1 (4.5%)	2 (4.2%)	>0.999
Minor complication			
Sinusitis (n = 67)	3 (13.6%)	3 (6.7%)	0.386
Epiphora (n = 69)	0 (0%)	0 (0%)	
Iatrogenic septal perforation (n = 69)	0 (0%)	3 (6.4%)	0.546
Deep vein thrombosis (n = 69)	0 (0%)	0 (0%)	
No adhesions (n = 66)	8 (36.4%)	15 (34.1%)	0.855
Local adhesiolysis (n = 66)	14 (63.6%)	29 (65.9%)	0.855
Surgical outcomes			
Endocrine status unchanged (n = 72)	18 (81.8%)	29 (58%)	0.082
Endocrine status improvement (n = 72)	3 (13.6%)	19 (38%)	0.082
Endocrine status worsening (n = 72)	1 (4.5%)	2 (4%)	0.082
Visual status unchanged (n = 67)	16 (84.2%)	31 (64.6%)	0.380
Visual status improvement (n = 67)	3 (15.8%)	16 (33.3%)	0.380
Visual status deterioration (n = 67)	0 (0%)	1 (2.1%)	0.380
No smell impairment (>6 months)(n = 69)	21 (95.5%)	1 (85.1%)	0.601
Hyposmia (>6 months) (n = 69)	1 (4.5%)	6 (12.8%)	0.601
Anosmia (>6 months) (n = 69)	0 (0%)	1 (2.15)	0.601
Surgical overall outcome			
Surgical success (n = 71)	11 (50%)	31 (63.3%)	0.293
Gross tumor resection (n = 64)	13 (68.4%)	29 (64.4%)	0.760
Endocrine remission (n = 56)	5 (33.3%)	26 (63.4%)	0.045
Adjuvant treatment			
Irradiation treatment (n = 71)	0 (0%)	6 (12.2%)	0.167
Overall Gy dosage (n = 5)		27.20 ± 5.40 26 [23–32]	

ASA = American society of Anesthesiology; CSF = cerebrospinal fluid; NSF = nasoseptal flap; DI = diabetes insipidus; categorical parameters are presented as N(%); continuous parameters are presented as mean ± standard deviation; median (50%) [interquartile range, IQR [25–75%]]. Bold: statistically significant values.

**Table 4:** Comparison of QOL Properties between those who underwent middle turbinate resection vs. middle turbinate preservation (n = 73).

Parameter (n = 73)	Middle Turbinate Preservation (n = 22)	Middle Turbinectomy (n = 51)	*p*-Value
Preoperative questionnaire scores			
Nasal-related QOL: SNOT-22 (n = 67)	28.32 ± 16.83 28.00 [10.00–40.00]	22.77 ± 17.24 16.50 [8.25–37.75]	0.170
Tumor-related QOL: Overall ASBQ	3.83 ± 0.86 3.94 [3.42–4.44]	4.16 ± 0.70 4.36 [3.74–4.71]	0.095
ASBQ subdomains			
Role of performance	3.78 ± 0.89 4 [3–4.37]	4.12 ± 0.83 4.28 [3.71–4.83]	0.099
Physical function	4.04 ± 0.91 4.12 [3.59–4.91]	4.39 ± 0.76 4.75 [4.12–5.00]	0.093
Vitality	3.61 ± 1.08 3.90 [2.67–4.37]	4.07 ± 0.94 4.17 [3.67–4.83]	**0.049**
Pain	3.48 ± 1.11 3.33 [2.92–4.42]	4.00 ± 1.02 4.33 [3.50–4.83]	0.055
Specific symptoms	4.18 ± 0.73 4.21 [3.96–4.71]	4.32 ± 0.56 4.43 [4.00–4.71]	0.479
Emotional state	3.49 ± 1.13 3.60 [2.47–4.50]	3.78 ± 1.02 4.00 [2.80–4.60]	0.313
2-month postoperative questionnaire scores			
Nose-related QOL: SNOT-22 (n = 48)	39.00 ± 30.29 38.00 [6.50–60.75]	17.42 ± 16.43 11.00 [5.50–29.00]	0.054
Tumor-related QOL: Overall ASBQ (n = 52)	3.68 ± 1.01 3.64 [2.92–4.76]	4.41 ± 0.55 4.63 [4.22–4.86]	0.025
ASBQ subdomains			
Role of performance (n = 52)	3.69 ± 0.89 3.57 [3.07–4.64]	4.40 ± 0.58 4.50 [4.00–5.00]	0.008
Physical function (n = 52)	3.81 ± 1.09 3.62 [3.12–4.87]	4.54 ± 0.57 4.87 [4.00–5.00]	**0.024**
Vitality (n = 51)	3.72 ± 1.02 3.50 [3.00–4.75]	4.45 ± 0.63 4.67 [4.12–5.00]	**0.027**
Pain (n = 51)	3.49 ± 1.08 3.33 [2.33–4.50]	4.33 ± 0.71 4.33 [4.00–5.00]	**0.014**
Specific symptoms (n = 51)	3.63 ± 1.12 3.71 [2.57–4.78]	4.35 ± 0.66 4.43 [4.11–4.87]	**0.046**
Emotional state (n = 51)	3.68 ± 1.19 4.00 [2.80–4.80]	4.31 ± 0.74 3.64 [2.92–4.76]	0.085
2–6-month (TP2) postoperative questionnaire scores			
Nose-related QOL: SNOT-22 (n = 33)	38.17 ± 24.03 37.00 [14.00–61.75]	24.13 ± 21.99 21.00 [7.50–37.00]	0.158
Tumor-related QOL: Overall ASBQ (n = 34)	3.96 ± 0.97 4.25 [3.00–4.75]	4.34 ± 0.61 4.52 [3.97–4.85]	0.364
ASBQ subdomains			
Role of performance (n = 34)	3.71 ± 1.05 3.57 [3.00–4.79]	4.30 ± 0.57 4.38 [4.00–4.86]	0.173
Physical function (n = 34)	4.23 ± 0.82 4.50 [3.44–4.91]	4.60 ± 0.52 4.81 [4.50–5.00]	0.257
Vitality (n = 33)	3.86 ± 1.13 4.33 [2.75–4.62]	4.31 ± 0.84 4.60 [4.00–5.00]	0.324
Pain (n = 33)	3.78 ± 1.01 3.83 [3.25–4.50]	4.25 ± 1.09 4.67 [3.67–5.00]	0.173
Specific symptoms (n = 33)	4.19 ± 1.01 4.64 [2.96–5.00]	4.26 ± 0.70 4.57 [3.86–4.71]	0.768
Emotional state (n = 33)	3.83 ± 1.15 4.10 [2.75–4.85]	4.09 ± 0.96 4.40 [3.40–4.80]	0.600
6-month-and-onward (TP3) postoperative questionnaire scores			
Nose-related QOL: SNOT-22 (n = 37)	26.10 ± 24.69 19.50 [9.75–36.75]	28.22 ± 19.93 26.00 [8.00–45.00]	0.625
Tumor-related QOL: Overall ASBQ (n = 41)	4.17 ± 0.88 4.44 [3.55–4.87]	4.15 ± 0.65 4.17 [3.67–4.76]	0.682
ASBQ subdomains			
Role of performance (n = 41)	4.13 ± 0.91 4.50 [3.32–4.96]	4.11 ± 0.81 4.43 [3.43–4.78]	0.767
Physical function (n = 41)	4.19 ± 0.99 4.69 [3.19–4.97]	4.22 ± 0.76 4.25 [3.62–5.00]	0.966
Vitality (n = 41)	4.01 ± 0.95 4.17 [3.42–4.92]	4.00 ± 0.97 4.50 [3.25–4.75]	0.899
Pain subdomain (n = 41)	4.05 ± 0.91 4.17 [3.42–4.92]	4.02 ± 0.87 4.33 [3.42–4.67]	0.854
Specific symptoms (n = 41)	4.39 ± 0.73 4.78 [3.68–5.00]	4.31 ± 0.54 4.57 [3.86–4.78]	0.372
Emotional state (n = 41)	4.10 ± 1.03 4.30 [3.65–5.00]	4.07 ± 0.83 4.20 [3.50–4.80]	0.724

Categorical parameters are presented as N(%); continuous parameters are presented as mean ± standard deviation; median (50%) [interquartile range, IQR [25–75%]]. SNOT-22: sinonasal quality of life tool; ASBQ: anterior skull base questionnaire. Bold: statistically significant values.

**Table 5:** Score difference over time between preoperative QOL scores at three different time points among the entire cohort (n = 73).

Parameter (n = 73)	Questionnaire Score (mean ± SD)	*p*-Value
2-month postop (TP1) scores versus pre-op		
Nose-related QOL: SNOT-22 (n = 44)	−0.11 ± 15.28 −2.50 [−8.00–6.75]	0.491
Tumor-related QOL: Overall ASBQ (n = 52)	0.12 ± 0.65 0.03 [−0.17–0.43]	0.308
ASBQ subdomains		
Role of performance (n = 52)	0.16 ± 0.78 0.06 [−0.29–0.71]	0.184
Physical function (n = 52)	0.05 ± 0.70 0.00 [−0.45–0.38]	0.824
Vitality (n = 51)	0.21 ± 0.77 0.17 [−0.33–0.67]	0.101
Pain (n = 51)	0.21 ± 1.03 0.00 [−0.33–0.67]	0.226
Specific symptoms (n = 51)	−0.16 ± 0.76 0.00 [−0.71–0.29]	0.162
Emotional state (n = 51)	0.46 ± 0.95 0.20 [0.00–1.20]	**0.001**
2–6-month postop (TP2) scores versus pre-op		
Nose-related QOL: SNOT-22 (n = 29)	2.74 ± 14.69 5.00 [−6.75–14.00]	0.294
Tumor-related QOL: overall ASBQ (n = 34)	0.09 ± 0.64 0.06 [−0.11–0.35]	0.166
Subdomain		
Role of performance (n = 34)	0.08 ± 0.79 −0.07 [−0.21–0.43]	0.723
Physical function (n = 34)	0.06 ± 0.63 0.00 [−0.13–0.25]	0.615
Vitality (n = 33)	0.21 ± 0.91 0.17 [−0.17–0.73]	0.201
Pain (n = 33)	0.17 ± 1.02 0.17 [−0.17–0.67]	0.085
Specific symptoms (n = 33)	−0.07 ± 0.68 0.00 [−0.36–0.36]	0.855
Emotional state (n = 33)	0.17 ± 1.02 0.00 [−0.50–0.90]	0.398
6-months-onward (TP3) scores versus pre-op		
Nose-related QOL: SNOT-22 (n = 35)	1.71 ± 18.00 1.00 [−8.00–11.00]	0.594
Tumor-related QOL: overall ASBQ (n = 41)	0.15 ± 0.71 0.12 [−0.16–0.45]	0.120
Role of performance (n = 41)	0.09 ± 0.89 0.00 [−0.29–0.58]	0.398
Physical function (n = 41)	−0.02 ± 0.78 0.00 [−0.40–0.38]	0.857
Vitality (n = 41)	0.15 ± 0.93 0.17 [−0.42–0.68]	0.286
Pain (n = 41)	0.25 ± 1.21 0.00 [−0.58–1.00]	0.299
Specific symptoms (n = 41)	0.17 ± 0.70 0.14 [−0.43–0.71]	0.155
Emotional burden (n = 41)	0.41 ± 1.09 0.20 [−0.28–1.20]	**0.035**

Categorical parameters are presented as N (%); continuous parameters are presented as mean ± standard deviation; median (50%) [interquartile range, IQR [25–75%]]. SNOT-22: sinonasal quality of life tool; ASBQ: anterior skull base questionnaire. Time points were defined as TP1: 2 months, TP2: 2–6 months, TP3: 6 months and more; QOL scores were compared to preoperative baseline. Bold: statistically significant values.

**Table 6:** The score difference between pre-op and post-op follow-up at three different time points (TP1:2 months, TP2:2–6 months, TP3:6 months and more) between patients with and those without middle turbinectomy and within each group (n = 73).

Parameter (n = 73)	Middle Turbinate Preservation	N	*p*-Value Within Group	Middle Turbinectomy	N	*p*-Value Within Group	*p*-Value Between Groups
2-month post-op (TP1) scores versus pre-op							
Nose-related QOL: SNOT22	7.00 ± 18.17 8.00 [−7.75–8.00]	8	0.917	−1.69 ± 0.00 −4.00 [−8.00–1.00]	36	0.214	0.179
Tumor-related QOL: overall ASBQ	0.03 ± 0.64 −0.01 [−0.57–0.42]	13	0.780	0.15 ± 0.68 0.04 [−0.09–0.46]	39	0.161	0.583
Role of performance	0.04 ± 0.65 0.02 [−0.51–0.68]	13	0.959	0.20 ± 0.82 0.09 [−0.29–0.71]	39	0.789	0.575
Physical function	0.03 ± 0.73 0.00 [−0.55–0.50]	13	0.387	0.05 ± 0.70 0.00 [−0.29–0.25]	39	0.209	0.924
Vitality	0.23 ± 0.83 0.33 [−0.42–0.58]	13	0.766	0.20 ± 0.76 0.00 [−0.33–0.83]	38	0.246	0.558
Pain	0.10 ± 0.88 0.00 [−0.50–0.67]	13	0.195	0.25 ± 1.08 0.00 [−0.33–0.67]	38	0.394	0.912
Specific symptoms	−0.40 ± 0.95 −0.14 [−1.29–0.43]	13	0.125	−0.08 ± 0.69 0.00 [−0.57–0.29]	38	0.006	0.346
Emotional state	0.38 ± 0.82 0.20 [−0.10–1.00]	13	0.362	0.49 ± 1.00 0.20 [0.00–1.25]	38	0.148	0.632
2–6-month post-op (TP2) scores versus pre-op							
Nose-related QOL: SNOT22	6.40 ± 14.50 6.00 [−5.50–18.50]	5	0.463	1.98 ± 14.92 3.50 [−6.87–12.75]	24	0.038	0.382
Tumor-related QOL: overall ASBQ	−0.12 ± 0.95 −0.22 [−0.75–0.43]	6	0.344	0.14 ± 0.57 0.08 [−0.05–0.38]	28	0.351	0.110
Role of performance	−0.13 ± 1.29 −0.26 [−0.79–0.43]	6	0.500	0.13 ± 0.66 0.00 [−0.14–0.43]	28	0.305	0.173
Physical function	−0.02 ± 0.87 −0.19 [−0.53–0.41]	6	0.686	0.08 ± 0.58 0.00 [−0.13–0.25]	28	0.058	0.159
Vitality	−0.12 ± 1.37 −0.25 [−1.35–0.71]	6	0.785	0.28 ± 0.80 0.17 [−0.17–0.80]	27	0.027	0.241
Pain subdomain	0.00 ± 1.12 −0.17 [−1.00–0.75]	6	0.528	0.21 ± 1.02 0.33 [0.00–0.67]	27	0.845	0.241
Specific symptoms	−0.17 ± 0.75 −0.21 [−0.82–0.46]	6	0.399	−0.05 ± 0.68 0.00 [−0.29–0.43]	27	0.211	0.508
Emotional state	−0.24 ± 0.78 −0.40 [−0.76–0.40]	6	0.223	0.26 ± 1.06 0.10 [−0.40–1.20]	27	0.475	0.205
6-months-onward (TP3) scores versus pre-op							
Nose-related QOL: SNOT-22	4.89 ±21.96 1.00 [−7.50–22.50]	9	0.209	0.61 ± 16.78 0.50 [−8.00–11.50]	26	0.285	0.616
Tumor-related QOL: overall ASBQ	0.28 ± 0.71 0.20 [−0.19–0.69]	12	0.238	0.10 ± 0.71 0.11 [−0.22–0.45]	29	0.782	0.543
Role of performance	0.28 ± 0.79 0.14 [−0.25–0.79]	12	0.964	0.01 ± 0.92 0.00 [−0.46–0.57]	29	0.819	0.488
Physical function	0.00 ± 0.69 −0.06 [−0.22–0.34]	12	0.254	0.14 ± 0.57 0.08 [−0.05–0.38]	29	0.602	0.944
Vitality	0.33 ± 0.92 0.25 [−0.29–0.69]	12	0.152	0.07 ± 0.94 0.17 [−0.50–0.67]	29	0.769	0.641
Pain	0.56 ± 1.23 0.33 [−0.25–1.58]	12	0.383	0.12 ± 1.19 0.00 [−0.67–0.83]	29	0.238	0.205
Specific symptoms	0.17 ± 0.68 0.07 [−0.14–0.54]	12	0.123	0.17 ± 0.72 0.14 [−0.50–0.71]	29	0.138	0.921
Emotional state	0.59 ± 1.13 0.40 [−0.15–1.15]	12	0.362	0.34 ± 1.08 0.20 [−0.38–1.30]	29	0.909	0.601

Categorical parameters are presented as N(%); continuous parameters are presented as mean ± standard deviation; median (50%) [interquartile range, IQR [25–75%]]. SNOT-22: sinonasal quality of life tool; ASBQ: anterior skull base questionnaire. Time points were defined as TP1: 2 months, TP2: 2–6 months, TP3: 6 months and more; QOL scores were compared to preoperative baseline. *p*-value between groups indicates comparison of score differences in each time point between middle turbinate preservation vs. resection. *p*-value within group indicates comparison between baseline pre-op score difference from a specific time point score.

**Table 7:** Linear mixed models adjusted to age, sex, intraoperative CSF leak, endocrine secretion status of tumor and size to compare the change in SNOT22 and ASBQ between patients undergoing EEA with and without middle turbinate resection.

Parameter (n = 15)	Time Point	*p*-Value for Interaction
Nose-related QOL: SNOT22	Preoperative scores	0.254
	2 months postop (TP1)	0.238
	2–6 months post-op (TP2)	0.284
	6 months onwards (TP3)	0.544
Tumor-related QOL: overall ASBQ	Preoperative scores	0.251
	2 months postop (TP1)	0.568
	2–6 months post-op (TP2)	0.824
	6 months onwards (TP3)	0.763
ASBQ subdomain		
Role of performance	Preoperative scores	0.218
	2-months postop (TP1)	0.552
	2–6 months post-op (TP2)	0.618
	6 months onwards (TP3)	0.559
Physical function	Preoperative scores	0.274
	2 months postop (TP1)	0.874
	2–6 months post-op (TP2)	0.988
	6 months onwards (TP3)	0.965
Vitality	Preoperative scores	0.187
	2 months postop (TP1)	0.832
	2–6 months post-op (TP2)	0.834
	6 months onwards (TP3)	0.528
Pain	Preoperative scores	0.193
	2 months postop (TP1)	0.606
	2–6 months post-op (TP2)	0.738
	6 months onwards (TP3)	0.318
Specific symptoms	Preoperative scores	0.796
	2 months postop (TP1)	0.123
	2–6 months post-op (TP2)	0.745
	6 months onwards (TP3)	0.950
Emotional state	Preoperative scores	0.431
	2-month postop (TP1)	0.664
	2–6 months post-op (TP2)	0.955
	6 months onwards (TP3)	0.747

Model were adjusted to age, sex, intraoperative CSF leak, endocrine secretion status of tumor (secreting or non-secreting adenoma), tumor size. QOL: quality of life. SNOT-22: sinonasal quality of life tool; MT: middle turbinate; CSF: cerebral spinal fluid; ASBQ: anterior skull base questionnaire. Time points were defined as TP1: 2 months, TP2: 2–6 months, TP3: 6 months and more.

## Data Availability

The data presented in this study are available on request from the corresponding author due to privacy and ethical restrictions.
